# An Act to Establish a Live-stock Commission, Etc. (Pennsylvania)

**Published:** 1894-10

**Authors:** 


### PENNSYLVANIA.

### AN ACT

TO ESTABLISH A LIVE-STOCK COMMISSION, TO REGULATE THE ERADICATION AND SPREAD OF CONTAGIOUS DISEASES, AND TO SECURE DAIRY INSPECTION.

SECTION 1. Be it enacted by the Senate and House of Representatives, etc.: That a Commission, known as the Live-stock Commission, be, and hereby is, established. Said Commission shall be composed of five members: three veterinarians, who shall be of good standing in their profession, graduates from a recognized veterinary college, and selected from among the list of members of the Pennsylvania State Veterinary Medical Association, the President of the State Board of Health, and a member of the State Board of Agriculture. They shall be appointed by the Governor of this Commonwealth, and shall serve continuously for a period of three years, or until their successors are appointed and duly qualified. Said Commissioners shall have power to make rules and regulations to execute the provisions of this Act, providing such rules and regulations do not conflict with the rules and regulations of this State or of the United States.

SECTION 2. Said Commission shall respectively take an oath of office to perform the duties of their office faithfully, shall immediately organize by electing one of their members as President, and shall forthwith proceed with the discharge of their duties as provided for in this Act. Their salary shall be fixed by the Governor.

SECTION 3. The Governor shall have power to revoke the appointment of one or more of the appointees for incompetency or continued neglect of duties. Vacancies accruing from this or for any other cause shall be refilled by the Governor as herein provided for.

SECTION 4. Said Commission shall maintain an office and hold meetings at the call of the President or of his two colleagues at such times and places as is most convenient for the performance of their duties.

A detailed record of all transactions and of all animals examined or destroyed shall be kept in said office for future reference.

SECTION 5. It shall be the duty of the said Commissioners to cause investigation to be made as to the existence of all contagious and infectious diseases, especially of tuberculosis, and such Commissioners, or their duly constituted deputies or agents, are hereby authorized to enter any premises or places, including dairies, slaughter-houses, stockyards, vessels, etc.. within any port or county in this State in or at which they have reason to believe any such disease or diseases exist, and to make search, investigation and inquiry in regard to the existence thereof; also are they hereby authorized and required to establish and maintain such quarantine of animals, places, dairies or localities as they may deem necessary to prevent the spread and transmission of such disease. They are also invested with power to destroy and dispose of such diseased animals as herein provided for.

SECTION 6. Said Commission shall also be authorized, at the request of the State Board of Health, a local Board of Health where such exists, and in all cities, towns and boroughs where no such local Board exists, then at the request of the Mayor, the Burgess or Burgesses, to appoint a local deputy, or more than one if necessary, who shall be called Dairy Inspectors, whose salary shall be fixed by such city, town or borough wherein the appointment is made. Such Inspectors, whose work shall be assisted by a local Board or by the State Board of Health, shall be under the authority, control and direction of the Live-stock Commission, whose duty it shall be to prepare, print and distribute to all producers and venders of milk, and to all Dairy Inspectors, general circulars for the uniform direction, guidance and control of the said Inspectors of dairies and their assistants.

SECTION 7. The Dairy Inspector and his assistants of any city, town or borough, in order to carry out the purposes of this Act, shall have the right at any and all times to enter upon or into the premises of any producer of milk. They shall carefully inspect, inquire into and report to the said Commission without delay upon all matters and things which they are directed and required by the said Commission to inquire into and report upon. And they shall from time to time examine all milch cattle of said milk producer for the discovery of tuberculosis or other infectious diseases among the same, using the manner and means of examination prescribed by the said Commission.

SECTION 8. Whenever any animal in a herd of milch cows or any other animal or aggregation of animals in any locality or place whatsoever examined under authority aforesaid by the said Commission, their deputies or Dairy Inspector or Inspectors, is by him or them discovered to be the subject of an infectious or contagious disease in any degree, the said animal shall be immediately branded or securely tagged and safely separated and kept apart from the rest of the herd; and it shall be unlawful for the carcass, the milk or flesh of said diseased animal to be sold, offered or exposed for sale, delivered or received. or purchased for sale by any person or persons whomsoever with the intent, purpose or probability that it may be the means of spreading such disease or become the food of man or domestic animal. Any violation of this section or any of them shall be punished by a fine of not less than two hundred dollars ($200), nor more than five hundred dollars ($500), together with imprisonment of not less than four months or more than one year.

SECTION 9. The sum of fifty thousand dollars ($50,000) is hereby appropriated for the purpose of providing and defraying the expense of carrying out the requirements of this Act, to wit: So long as there is any money in the State Treasury available for the purpose, whenever any animal is found by the said Commission, their deputies or Dairy Inspector to be the victim of tuberculosis or other contagious disease in any degree, in addition to the requirements of Section 8, a jury of three reputable citizens, well versed in the value of cattle, shall be appointed without delay, one of whom may be appointed by the said owner, but if he neglect or refuse to exercise his right, then by the Commission or said Dairy Inspector, where such exists; one by the said Commission or Dairy Inspector, where such exists, and one to be chosen by the other two; and the said jury, after having been sworn (and the Inspector or member of the Commission or his deputy is hereby authorized to administer oaths for this purpose), shall impartially appraise the actual cash value, in its diseased condition, of the said animal; and the surroundings of the herd shall be given due consideration in said valuation, and no animal shall be assessed, for the purpose of this Act, at a higher sum than forty dollars ($40).

The said Commission or Inspector shall thereupon cause the said animal discovered to be the victim of tuberculosis or other infectious or contagious disease to be immediately slaughtered and speedily disposed of, according to the directions of the said Commission, in such a manner that no part of the carcass be consumed, or be liable to be consumed, as food for man or domestic animal. If the autopsy show that the said animal was the victim of tuberculosis, or of any other highly dangerous, contagious or infectious disease, in that case the owner of said slaughtered animal shall be paid out of the said fifty thousand dollars ($50,000) a sum equal to one-third the said assessed value. If, however, the autopsy of the slaughtered animal reveal the presence of no infectious or other disease, dangerous or harmful to health through consumption of the flesh or milk thereof, then and in that case the owner of the said slaughtered animal shall be paid out of the said fund of fifty thousand dollars ($50,000) an amount equal to the said assessed value of said animal; or, in lieu thereof, at the option of the owner, the carcass shall be turned over to the said owner, to be disposed of by him as beef or otherwise.

SECTION 10 It shall be unlawful for any person to interfere with any Commission, Inspector, deputy or assistant herein provided for in the performance of his duties under this Act. A violation of the provisions of this section shall be punished in the name provided for in Section 8 of this Act.

SECTION 11. This Act shall go into effect on the first day of June, 1895, and all Acts or provisions of Acts inconsistent herewith are hereby repealed.

